# Supplementary figures and images for: Improving health care from the bottom up: Factors for the successful implementation of kaizen in acute care hospitals
Source: PLoS One. 2021 Sep 10;16(9):e0257412. doi: 10.1371/journal.pone.0257412 (PMC8432859; doi:10.1371/journal.pone.0257412)

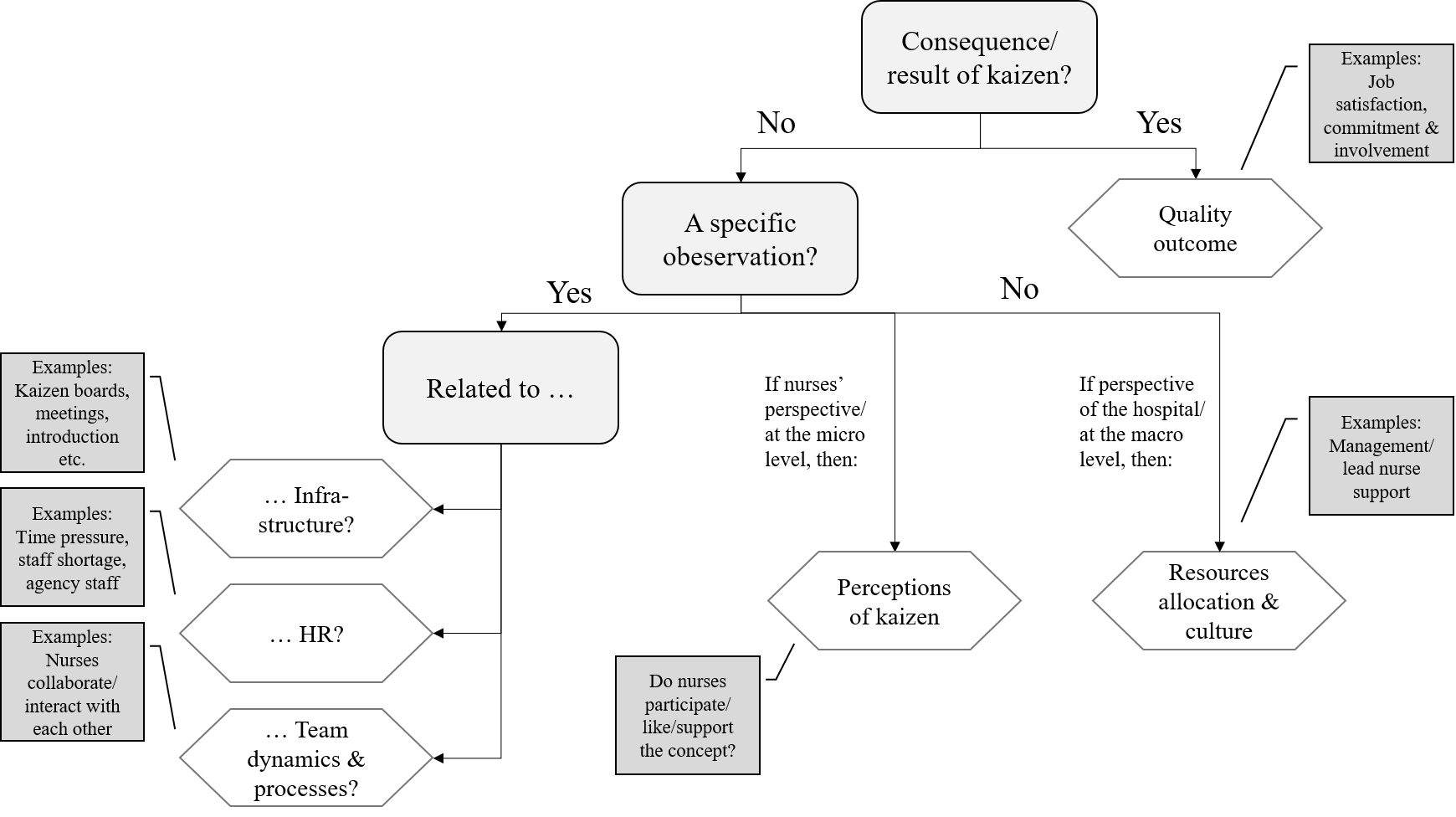

Supplement: S1 Fig — (PNG) [file pone.0257412.s001.png]
