# Supplementary material for: Improving health care from the bottom up: Factors for the successful implementation of kaizen in acute care hospitals
Source: PLoS One. 2021 Sep 10;16(9):e0257412. doi: 10.1371/journal.pone.0257412 (PMC8432859; doi:10.1371/journal.pone.0257412)
Supplement: S1 Table — (DOCX) [file pone.0257412.s002.docx]

**S1 Table:** The interview guide, which we used for the semi-structured interviews.

| Interview ID: | Hospital ID: | Interviewed person: | Date: |
| --- | --- | --- | --- |

| **Section I: Introduction & Consent** | |
| --- | --- |
| **Consent to participate:**   - **Yes** - **No** | *The study design and interview guide were approved by the responsible ethics committee of our university. I would like to record the interview so that it can be transcribed afterwards and also for the sake of transparency. We are obliged to abide by the data protection regulations of our university. The transcribed interviews will be stored on servers of our university and will be deleted twelve months after the publication of the manuscript. Please do not hesitate to tell me if you* *if you do not want the interview to be recorded.* |

| **Section II. Background information** | |
| --- | --- |
| *How old are you?* |  |
| *How long have you been working at this hospital?* |  |
| *What is your position and which tasks are your responsible for?* |  |
| *Please describe briefly what your usual workday at the hospital looks like by giving some examples.* |  |

| **Section III. Questions about participation in the work processes associated with kaizen, structure & daily routine** | | |
| --- | --- | --- |
| **Q1*: "The staff in your ward is encouraged to report problems and make suggestions for improvement. Is this correct?***  ***Can you please describe what this looks like in practice?"*** | | |
| **Auxiliary questions** | | |
| *Main auxiliary questions* | | *Secondary auxiliary questions* |
| *What kinds of suggestions have been made?* | |  |
| *Can you describe the conversations with your colleagues?* | | - *Are all employees involved? How?* - *How much are you involved in these discussions? Why?* - *Do you think that, for example, twice a month is enough? Why or why not?* |
| *Do you have specific responsibilities within the kaizen management technique?* | | - *How do you exercise these responsibilities?* - *If not, why?* - *How would you judge your involvement in kaizen? Why?* |
| *Who makes the final decisions?* | | - *Do you consider this fair?* - *Are there other people who would be more suited to make these decisions?* |
| *What is the impact of kaizen on the way your ward operates?* | | - *What is your personal attitude towards kaizen?* - *What do you associate with the word "kaizen" when you hear it?* |
| *Do you have any previous experience with kaizen?* | | - *What was your initial experience when you started to work at the hospital?* - *Were you skeptical at the beginning?* - *Did something change afterwards? Why?* |
| *Have you already made any suggestions?* | | - *Why?* - *What makes you think that a particular issue should be reported?* - *Does the number of suggestions made by your colleagues have any influence on you?* |
| *Notes:* | | |
| **Q2: *"Could you give an example of a suggestion you have made for solving a specific problem?"*** *(If not, why?)* | | |
| **Auxiliary questions** | | |
| *Main auxiliary questions* | *Secondary auxiliary questions* | |
| *Then what happened? Did your kaizen suggestion have any consequences?* | - *How was it implemented?* - *What did you think when you noticed that your solution led to some visible improvements?* - *Has it ever been the case that your solution was perceived to be inefficient or inconvenient after it was implemented? What happened then?* | |
| *Why did you make this suggestion?* | - *Do you talk regularly with your colleagues about problems? Why?* - *And with your supervisors? Why?* | |
| *Do you remember how you felt when your suggestion was considered/not considered?* | - *Why did you feel that way?* - *Did that feeling affect your motivation to make further suggestions? Why?* | |
| *How was this suggestion perceived by your supervisor?* | - *Is your supervisor’s opinion of your suggestion more important to you than your colleagues’ opinions? Why?* | |
| *Would the way and amount you participate change if you had no access to the kaizen tools?* | - *How?* - *Why?* | |
| *Notes:* | | |
| **Q3*: "Does participating in kaizen affect your attitude towards the hospital (as an employer)?”*** | | |

| **Auxiliary questions** | |
| --- | --- |
| *Main auxiliary questions* | *Secondary auxiliary questions* |
| *Why?* | - *Could you be more specific about your feelings?* - *How do these feelings affect your work?* |
| *Do your suggestions address job-related issues? What about patient satisfaction?* | - *Why?* - *How important is patient satisfaction to you? What about the satisfaction of employees? Why?* |
| *Does the opportunity to express your opinion have any influence on your willingness to continue to work for the hospital?* | - *In what way? What role does kaizen play compared to other factors when considering whether or not to stay at the hospital?* - *What about job satisfaction? Why?* |
| *In what way could kaizen improve the relationship with your colleagues?* | - *And with your supervisor?* |
| *Notes:* | |

| **Section IV. Questions about desired participation in the work process** | |
| --- | --- |
| **Q4: "*How important is it for you to express your own opinion?”*** | |
| **Auxiliary questions** | |
| *Main auxiliary questions* | *Secondary auxiliary questions* |
| *Why is it important?* |  |
| *How do you feel when you share your opinion about a specific issue in front of other people?* | - *Do you find this useful? Why?* |
| *What factors motivate you to express your opinion?* | - *Why is/are this/these factor(s) so important?* - *Do you encounter this/these factor(s) in your workplace?* |
| *Are there any disadvantages that you associate with the possibility of expressing your opinion?* | - *Which ones?* - *Could these disadvantages sometimes discourage you from expressing your opinion? Why? Has this ever happened?* |
| *Do you think that you express your opinion differently at work than you do in private?* | - *Why?* |
| *Notes:* | |
| **Q5: *"How important is the opinion of the nursing staff in your job?"*** | |
| **Auxiliary questions** | |
| *Main auxiliary questions* | *Secondary auxiliary questions* |
| *In your opinion, why do you have the opportunity to make kaizen suggestions?* | - *May physicians also make kaizen suggestions?* - *Do you consider this fair? Why?* |
| *Notes:* | |
| **Q6: "** **Compared to your participation so far, would you like to participate more, less, or the same amount in the future?*”*** | |
| **Auxiliary questions** | |
| *Main auxiliary questions* | *Secondary auxiliary questions* |
| *If more: What do you think the reasons/factors are that prevent you from participating more?* |  |
| *If more: Who do you think should have the responsibility to promote participation?* |  |
| *If less: What are the reasons that make you participate as much as you do?* |  |
| *If equally: What do you think about the freedom to make suggestions only if necessary?* | - *Why?* |
| *Are there any aspects of the approach that you would like to change?* |  |
| *Notes:* | |

| **Section V. Questions about organizational support** | |
| --- | --- |
| **Q7: *"How would you evaluate the general support of hospital management in your daily job?”*** | |
| **Auxiliary questions** | |
| *Main auxiliary questions* | *Secondary auxiliary questions* |
| *Can you describe the support you receive?* | - *How do you feel when you receive this support?* - *Has it ever happened that you needed support for a specific problem, but nobody from the management team was willing to help you?* |
| *Do you feel part of the hospital?* | - *Why?* |
| *How do you contribute to the success of the hospital?* | - *What is your personal contribution? Could you do even more?* |
| *How important do you think is it to your supervisor that you work independently?* | - *Why?* - *Can you work independently?* |
| *Notes:* | |

| **Section VI. End of the interview** |
| --- |
| *Is there anything else you would like to add?* |
| Word of thanks. |
