# Supplementary material for: Improving health care from the bottom up: Factors for the successful implementation of kaizen in acute care hospitals
Source: PLoS One. 2021 Sep 10;16(9):e0257412. doi: 10.1371/journal.pone.0257412 (PMC8432859; doi:10.1371/journal.pone.0257412)
Supplement: S4 Table — (DOCX) [file pone.0257412.s005.docx]

**S4 Table. Overview of the decision rules we developed to specify the scope of the categories we applied to perform the qualitative content analysis of the interviews.**

| **Category** | **Sub-category** | **Decision rule** |
| --- | --- | --- |
| Nurses' perspectives | Nurses participate in kaizen by making suggestions and/or implementing ideas | If possible, always choose a category more specific than “Nurses’ perspectives”. |
|  | Nurses support the use of kaizen at the hospital |  |
|  | Other general or specific perceptions of kaizen and its importance |  |
| Job commitment and satisfaction | Kaizen increases commitment to the hospital | If a quote can be assigned to both a specific category (i.e., “HR”, “Infrastructure”, “Team dynamics and processes”) and to one of the other more abstract categories (i.e., “Resources allocation & culture”, “Quality outcome”), then also choose the specific one. |
|  | Kaizen increases overall job satisfaction |  |
| Team dynamics and processes | Staff fit with the program either through commitment to the program vision and/or experience and skills needed for successful implementation | “Team dynamics and processes” category is not about the relation between nurses and physicians/the management of the hospital. For that reason, choose “Resources allocation & culture” if the topic of the quote is related to physicians/the management of the hospital. |
|  | Team work, coordination, and cohesion in terms of how well hospital staff work together, support each other, and create a collaborative work environment |  |
|  | There is a clear pre-defined process/structured way, in which kaizen works | Select if a (detailed) process is described. |
| Infrastructure availability and adoption | Infrastructure needed for the sustainable implementation of kaizen is available and accessible, e.g. introductory course, dashboards, and regular meetings |  |
|  | Nurses make use of the kaizen infrastructure provided |  |
| Human resources and staffing | Constrains with existing staff, e.g. short-staffed and/or there is not enough time to include kaizen in their work routine |  |
|  | High staff turnover and/or extended use of external staff |  |
| Resource allocation and culture | The adequacy of resources dedicated to program implementation and/or achieving sustainable results | If a quote can be assigned to both a specific category (i.e., “HR”, “Infrastructure”, “Team dynamics and processes”) and to one of the other more abstract categories (i.e., “Resources allocation & culture”, “Quality outcome”), then also choose the specific one. |
|  | Management shows general support and/or encourages adoption of kaizen with persistence | Management = Managers AND/OR (Chief) Physicians |
|  | The management has established a culture, which promotes an open dialog and/or tolerates failure | Management = Managers AND/OR (Chief) Physicians |
