# Supplementary material for: Improving health care from the bottom up: Factors for the successful implementation of kaizen in acute care hospitals
Source: PLoS One. 2021 Sep 10;16(9):e0257412. doi: 10.1371/journal.pone.0257412 (PMC8432859; doi:10.1371/journal.pone.0257412)
Supplement: Attachment — Submitted filename: Response to Reviewers.docx [file pone.0257412.s007.docx]

# Rebuttal letter accompanying the second revision of our manuscript entitled “Improving health care from the bottom up: Factors for the successful implementation of kaizen in acute care hospitals”

Dear Editor, dear Reviewers, dear Editorial Team,

On behalf of all authors of the paper, I would like to thank you for providing your thoughtful comments on the revised version of our paper. We have carefully revised the manuscript accordingly, reorienting the section labeled “Limitations and further research” to the context of our qualitative study. We believe that our manuscript has benefited significantly as a result.

Below please find our detailed point-by-point replies to your comments.

# Journal requirements:

Please review your reference list to ensure that it is complete and correct. If you have cited papers that have been retracted, please include the rationale for doing so in the manuscript text, or remove these references and replace them with relevant current references. Any changes to the reference list should be mentioned in the rebuttal letter that accompanies your revised manuscript. If you need to cite a retracted article, indicate the article’s retracted status in the References list and also include a citation and full reference for the retraction notice.

Authors’ response: Thank you for this comment. None of the references we cite in the paper has been retracted. After reviewing the complete reference list, we made a few minor changes mentioned in R2 Table 1. All changes have been highlighted in red on a grey background. The “Identifier Revision 1” (ID_R1) stands for the reference number during the first revision round, whereas the second identifier (ID_R2) stands for the updated reference numbers (during this revision round):

**R2 Table 1. An overview of all changes to the reference list.**

| **ID_R1** | **ID_R2** | **Author(s)** | **Note** | **Revised reference (if needed)** |
| --- | --- | --- | --- | --- |
| 1 | 1 | Imai M. Kaizen | No changes needed |  |
| 2 | 2 | Bhuiyan N, Baghel A. | No changes needed |  |
| 3 | 3 | Knechtges P, Decker MC | No changes needed |  |
| 4 | 4 | Mazzocato P, Stenfors-Hayes T, von Thiele Schwarz U, Hasson H, Nyström ME. | No changes needed |  |
| 5 | 5 | Vera A, Kuntz L | No changes needed |  |
| 6 | 6 | Bailey C, Madden A, Alfes K, Fletcher L. | No changes needed |  |
| 7 | 7 | Glew DJ, O’Leary-Kelly AM, Griffin RW, van Fleet DD | No changes needed |  |
| 8 | 8 | Radnor Z, Boaden R | (1) Title corrected (by adding a question mark) as suggested on the publisher's website: https://www.tandfonline.com/doi/abs/10.1111/j.1467-9302.2008.00610.x?journalCode=rpmm20 (accessed August 11, 2021); (2) We have found a DOI, but did not add it to the reference list, because it was not active: DOI: 10.1111/j.1467-9302.2008.00610.x | Radnor Z, Boaden R. Lean in Public Services—Panacea or Paradox? Public Money & Management. 2008; 28:3–7. |
| 9 | 9 | Comtois J, Paris Y, Poder TG, Chaussé S. | Page range was incomplete | Comtois, Jonathan, et al. « L'approche Kaizen au Centre hospitalier universitaire de Sherbrooke (CHUS) : un avantage organisationnel significatif », Santé Publique, vol. 25, 2013, pp. 169-77. |
| 10 | 10 | Iannettoni MD, Lynch WR, Parekh KR, McLaughlin KA | No changes needed |  |
| 11 | 11 | D'Andreamatteo A, Ianni L, Lega F, Sargiacomo M | No changes needed |  |
| 12 | 12 | Filser LD, da Silva FF, Oliveira OJ de | Author's name corrected ("de Oliveira" instead of "Oliveira") | Filser LD, da Silva FF, de Oliveira OJ ~~de~~. State of research and future research tendencies in lean healthcare: a bibliometric analysis. Scientometrics. 2017; 112:799–816. doi: 10.1007/s11192-017-2409-8. |
| 13 | 13 | Jacobson GH, McCoin NS, Lescallette R, Russ S, Slovis CM | No changes needed |  |
| 14 | 14 | New S, Hadi M, Pickering S, Robertson E, Morgan L, Griffin D, et al. | No changes needed |  |
| 15 | 15 | Edmondson AC | No changes needed |  |
| 16 | 16 | Young T, Brailsford S, Connell C, Davies R, Harper P, Klein JH | No changes needed |  |
| 17 | 17 | Jørgensen F, Boer H, Gertsen F | No changes needed |  |
| 18 | 18 | De Pietro C, Camenzind P, Sturny I, Crivelli L, Edwards-Garavoglia S, Spranger A, et al. | We have adopted the citation suggested by the World Health Organization (cf. https://apps.who.int/iris/handle/10665/330252; accessed August 11, 2021) | World Health Organization. Regional Office for Europe, European Observatory on Health Systems and Policies, De Pietro, Carlo, Camenzind, Paul, Sturny, Isabelle. et al. (‎2015)‎. Switzerland: health system review. World Health Organization. Regional Office for Europe. https://apps.who.int/iris/handle/10665/330252. |
| 19 | 19 | Farsi M, Filippini M. | No changes needed |  |
| 20 | 20 | Hauser T, Salgado-Thalmann E, Bachmann M | We have added the editor (i.e., Federal Office of Public Health), as well as the follwoing access link: https://spitalstatistik.bagapps.ch/data/download/kzp18_publication.pdf?v=1592291836 | Hauser T, Salgado-Thalmann E, Bachmann M. Health insurance statistics. Key indicators of the Swiss hospitals 2018. Federal Office of Public Health 2020 [cited 6 May 2021]. Available from: https://spitalstatistik.bagapps.ch/data/download/kzp18_publication.pdf?v=1592291836. |
| 21 | 21 | Lukas CV, Holmes SK, Cohen AB, Restuccia J, Cramer IE, Shwartz M, et al. | No changes needed |  |
| 22 | 22 | Corley KG, Gioia DA | No changes needed |  |
| 23 | 23 | Bansal P, Corley K | No changes needed |  |
| 24 | 24 | Becker TE, Billings RS, Eveleth DM, Gilbert NL | No changes needed |  |
| 25 | 25 | Fedor DB, Caldwell S, Herold DM | No changes needed |  |
| 26 | 26 | Meyer JP, Allen NJ | No changes needed |  |
| 27 | 27 | Scott‐Ladd B, Travaglione A, Marshall V | No changes needed |  |
| 28 | 28 | Gill P, Stewart K, Treasure E, Chadwick B | No changes needed |  |
| 29 | 29 | Hirslanden | We have now added the place of publication of the hospital statistics report provided by the Hirslanden hospitals. | Hirslanden. Hospital Statistics. Fiscal Year 1.4.2017-31.3.2018. Glattpark, Switzerland; 2018. |
| 30 | 30 | Patton MQ | No changes needed |  |
| 31 | 31 | Sullivan JL, Adjognon OL, Engle RL, Shin MH, Afable MK, Rudin W, et al. | No changes needed |  |
| N/A | 32 | Lincoln YS, Guba EG | Added during the revision of the limitations section |  |
| 32 | 33 | Herzberg F, Mausner B, Snyderman BB. | No changes needed |  |
| 33 | 34 | Bassett‐Jones N, Lloyd GC | Title corrected (by adding a question mark) | Bassett‐Jones N, Lloyd GC. Does Herzberg's motivation theory have staying power? Journal of Mgmt Development. 2005; 24:929–43. doi: 10.1108/02621710510627064. |
| 34 | 35 | Wong CA, Spence Laschinger HK, Cummings GG | No changes needed |  |
| 35 | 36 | Drotz E, Poksinska B | No changes needed |  |
| 36 | 37 | Collar RM, Shuman AG, Feiner S, McGonegal AK, Heidel N, Duck M, et al. | No changes needed |  |
| 37 | 38 | Zaheer S, Ginsburg L, Chuang Y-T, Grace SL | No changes needed |  |
| 38 | 39 | Gagne M, Koestner R, Zuckerman M | (1) Author's name corrected ("é" instead of "e"); (2) Title corrected (by leaving out the superfluous character "1") | Gagne~~e~~é M, Koestner R, Zuckerman M. Facilitating Acceptance of Organizational Change: The Importance of Self-Determination~~1~~. J Appl Social Pyschol. 2000; 30:1843–52. doi: 10.1111/j.1559-1816.2000.tb02471.x |
| 39 | 40 | Clark DM, Silvester K, Knowles S | No changes needed |  |
| 40 | 41 | Simon RW, Canacari EG | No changes needed |  |
| 41 | 42 | Robertson E, Morgan L, New S, Pickering S, Hadi M, Collins G, et al. | No changes needed |  |
| 42 | 43 | Brännmark M, Holden RJ | No changes needed |  |
| N/A | 44 | Braun V, Clarke V | Added during the revision of the limitations section |  |
| N/A | 45 | Nowell LS, Norris JM, White DE, Moules NJ. | Added during the revision of the limitations section |  |
| N/A | 46 | Tobin GA, Begley CM | Added during the revision of the limitations section |  |
| 43 | 47 | Palinkas LA, Horwitz SM, Green CA, Wisdom JP, Duan N, Hoagwood K | No changes needed. |  |
| 44 | 48 | Fisher RJ, Katz JE | No changes needed |  |
| 45 | N/A | Zuckerman M | Removed during the revision of the limitations section |  |
| 46 | N/A | Diefenbach T. | Removed during the revision of the limitations section |  |
| 47 | N/A | Hunter DJ | Removed during the revision of the limitations section |  |
| 48 | N/A | Radnor ZJ, Holweg M, Waring J | Removed during the revision of the limitations section |  |

# Response to Reviewers

Reviewer #4: The authors have sufficiently addressed the reviewers’ comments. The manuscript is well-written and articulate. The authors have provided sufficient information including additional supplementary tables that have enriched the methods and findings of the manuscript. However, the limitation and further research section sounds like discussion of limitation for a quantitative rather than a qualitative research. The authors should revise the section with a view of clearly highlighting the trustworthiness of the study.

Authors’ response: Thank you for making us aware of this. We have carefully revised the section as follows:

1. We specify how we aimed to keep a self-critical account of our research by describing the extent to which our study meets the trustworthiness criteria suggested by Lincoln and Guba (1985) and illustrated by Nowell, et al. (2017). In so doing, we now highlight the rigor of our study, providing researchers with the opportunity to evaluate the trustworthiness of the research process (pages 27-28, lines 534-538):

*“[…] to help establish the trustworthiness of our qualitative research methods and results [32,45], we sought to ensure dependability by providing thorough and transparent documentation of our research interest, methodological choices, and qualitative results in the manuscript and its supporting information [46].”*

Additionally, we have now demonstrated that we have adhered to Braun and Clarke’s (2006) recommendations for familiarizing oneself with the qualitative data (page 27, lines 531-534):

*“[…] these researchers [KS and RB] had the chance to familiarize themselves with the data [44] by reading the transcribed interviews, having a series of discussions with the interviewer (CP), and receiving an introduction to the concept of kaizen and its principles by a team of medical experts (AG, KH, BT).”*

2. Another aspect of our revision of the limitations section is that we consolidated the limitations related to rigor and trustworthiness—and to transferability in particular—into two major groups of concern. The first group relates to how we chose to select the hospitals (page 28, lines 540-541):

*“First, the two acute care hospitals we examined in this study were private and profit-orientated. […]”*

The second group of limitations is related to the sampling technique we used to conduct the on-site interviews (page 28, lines 554-557):

*“While the purposive technique we used to select the interviewees enabled us to gain deep insight into the work environment of both hospitals, it may also have led us to place a disproportionate amount of attention to some experiences the nurses had had with kaizen. […]”*

By providing these detailed descriptions, we aim to help readers who seek to transfer our findings to another context to judge the transferability of our findings (Lincoln and Guba, 1985).

3. We now use language that is more positive. For example (page 28, lines 553-554):

*“Data collection is a second factor that should be considered when interpreting the findings of this study and judging their transferability.”*

4. We also streamlined the section to enhance its readability and relevance by deleting the following passages:

*“[…] our analysis relied on self-reported, retrospective, and cross-sectional data, which may have decreased the reliability of our results. […] It is also conceivable that some respondents may have tended to take credit for successes while denying responsibility for failure, leading to so-called self-serving bias [45].”*

*“Indeed, interviewees not being a reliable source of information—because of unconscious biases or conscious attempts to mislead the interviewer—is a common methodological problem in qualitative empirical research [46].”*

*“In addition, health care workers are more likely to talk openly about problems if their work unit and organization have psychologically safe work climates [15]. Thus, ironically, higher-performing units may look worse based on self-reported data simply because interviewees were more willing to speak their mind.“*

*“Lastly, and more generally, questioning the idea of implementing kaizen in health care was not the object of this study, although translating managerial concepts from industry into complex service industries like health care may not be an appropriate endeavor in the first place [49], especially at the systems level [50].”*

5. To demonstrate in a more convincing way that the limitations discussed do not pose a major limitation to our results we highlight the credibility of our research by adding the following passage (page 29, lines 567-573):

*“However, we do not expect reporting biases to have distorted our findings substantially because most nurses were not overly shy in criticizing hospital policies or the behaviors of their supervisors. By giving us an intimate look into their working place, the nurses enabled us not only to capture and explore their perceptions of kaizen, but also to realize that this managerial technique—no matter how beneficial it can be in some situations for both employee and organization—is not necessarily a panacea for all problems and aspirations managers may have.“*

6. In order to highlight the trustworthiness of our approach, we added the following sentence to the Methods section (page 11, lines 210-212):

*“In the next step, to enhance the credibility of analysis [32], two researchers (KS and RB) used the OTM framework to analyze the interviews.”*

# Minor editorial changes:

Finally, we made two changes to the Results sections to remove the following minor inconsistencies:

1. It should be “Hospital B”, not “Hospital 2” in (page 20, lines 378-379):

*“At the beginning it [kaizen] has an effect – I would say for about […] 4 weeks, or even only for 10 days, and then a lot, not everything, but a lot is forgotten’ (Hospital B/28)”*

2. We completed this sentence by adding the authors’ names (page 20, lines 385-386):

*“Noting that the study of kaizen has focused so far on success stories, D'Andreamatteo, et al. [11] and Filser, et al. [12] advocate learning from examples of less effective implementation.”*

We look forward to receiving your reply. Thank you very much for your time and consideration.

Yours faithfully,

The corresponding author on behalf of all authors of the paper
